# Comparison of Clinical and Radiological Improvement Between the Modified Trephine and High-speed Drill as Main Osteotomy Instrument in Pedicle Subtraction Osteotomy

**DOI:** 10.1097/MD.0000000000002027

**Published:** 2015-11-13

**Authors:** Hui Wang, Lei Ma, Dalong Yang, Di Zhang, Yong Shen, Wenyuan Ding

**Affiliations:** From the Department of Spine Surgery, The Third Hospital of HeBei Medical University, Shijiazhuang, China.

## Abstract

High-speed drill is the main osteotomy instrument in pedicle subtraction osteotomy (PSO) currently. Considering the long duration of surgery, the large amount of blood loss, and the high incidence of neurovascular injury, the osteotomy procedure is challenging. Use of trephine for the osteotomy displays high efficiency by shortening surgery time and reducing blood loss in anterior cervical corpectomy and fusion. However, the potential risk of neurological injury is high. We modified the trephine by adding locking instrument, when the serrated top of the trephine reaches the tip of the probe; the locking instrument on the probe restricts the trephine and improves security during the osteotomy procedure.

The aim of this study was to compare the clinical and radiological improvement between the modified trephine and high-speed drill as main osteotomy instrument in PSO.

From February 2009 to 2013, 50 patients with severe thoracolumbar kyphotic deformity caused by old compressive vertebrae were prospectively reviewed. All patients were randomly assigned to the experimental group (27 patients received PSO with modified trephine) and the control group (23 patients received PSO with high-speed drill). The clinical records were reviewed and compared for surgical time, operative blood loss, functional improvement (Oswestry Disability Index), and pain relief (visual analog scale). The radiological records were reviewed and compared for correction of kyphotic deformity postoperatively and correction loss at 2-year follow-up.

All patients successfully finished the PSO procedure, and got satisfactory kyphotic deformity correction and overall function improvement. The surgery time was shorter in the experimental group than that in the control group (132.7 ± 12.6 vs 141.7 ± 16.7 min; *P* = 0.03). No significant difference was found in blood loss (882.9 ± 98.9 mL vs 902.2 ± 84.9 mL; *P* = 0.47) or correction of the kyphotic angle (33.4 ± 3.4° vs 32.1 ± 2.5°, *P* = 0.13) postoperatively between the 2 groups. At 24-month follow-up, no difference was discovered in loss of the correction (4.9 ± 1.6° vs 4.5 ± 1.6°; *P* = 0.42), change of Oswestry Disability Index (49.4 ± 6.2% vs 48.2 ± 4.2%; *P* = 0.44), or in back pain relief (6.2 ± 1.4 vs 6.4 ± 1.2 min; *P* = 0.51) between the 2 groups. No internal fixation related complication occurred and bony fusion was detected in lateral X-ray in all patients. In the control group, 2 patients had transient nerve root deficit, 14 patients at 3-month follow-up and 3 patients at 2-year follow-up experienced graft donor site morbidity, and pain killer medicine was always required.

In conclusion, the modified trephine obviously shortens surgery time, and prevents graft donor site morbidity when compared to a high-speed drill. The learning curve for using the modified trephine in PSO procedure is short.

## INTRODUCTION

Severe thoracolumbar kyphosis may develop secondary to fracture, healed tuberculous infection, and congenital malformation.^1–5^ Irrespective of the etiology, the rigid kyphotic deformity requires surgical intervention to prevent progressive deterioration in sagittal imbalance and in neurologic function.^6–9^ Pedicle subtraction osteotomy (PSO), first described by Thomson, is an effective method for correcting the kyphotic deformity, and about 30° correction can be achieved by osteotomy at 1 level.^10–13^ Both the curette and osteotome are traditional osteotomy instruments, and are used in PSO to make a wedge osteotomy space within the target vertebrae.^14^ The volume of bone harvested by curette and osteotome is small, repetitive manipulation is required, making the osteotomy procedure sophisticated.^15–17^ Currently, high-speed drill is the primary osteotomy instrument in the osteotomy procedure; it allows easy manipulation, controls bleeding effectively, and has been proven to be safer than curette.^18^ However, the high incidence of neurological complications, the large amount of blood loss, and the long duration of surgery make the PSO a demanding procedure.^19–21^

**FIGURE 1:**
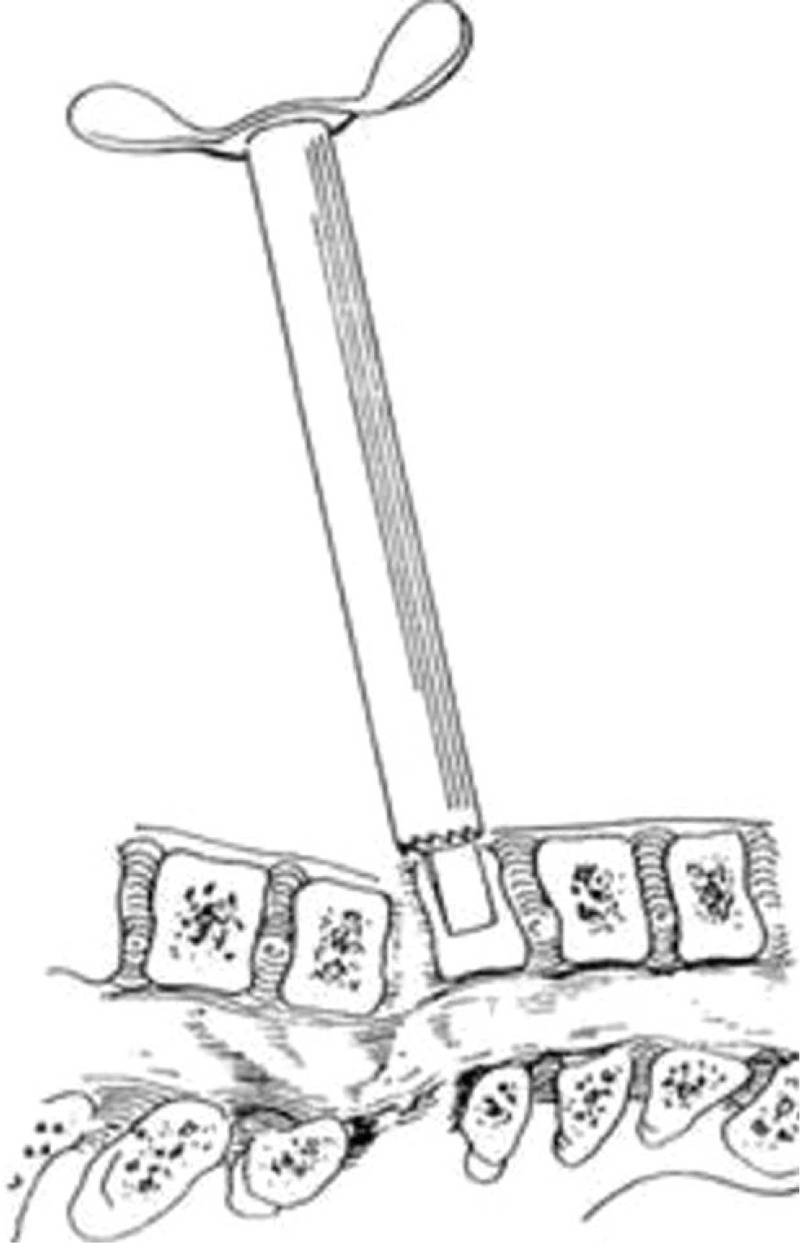
The majority of cervical vertebrae can be harvested by 1 manipulation of trephine in ACCF. ACCF = anterior cervical corpectomy and fusion.

**FIGURE 2:**
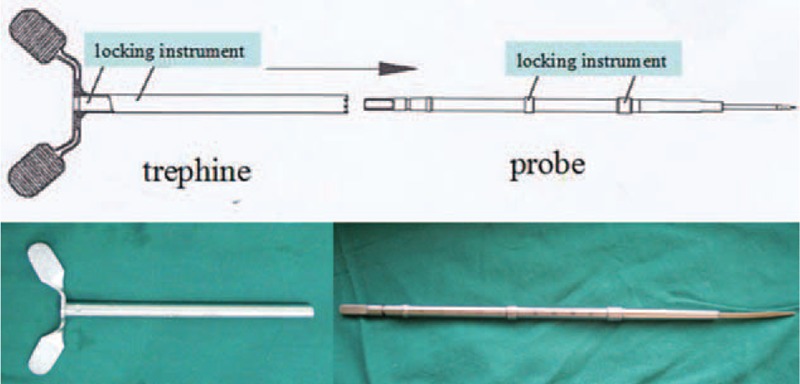
The modified probe and trephine, with restricted instruments on both of them.

**FIGURE 3:**
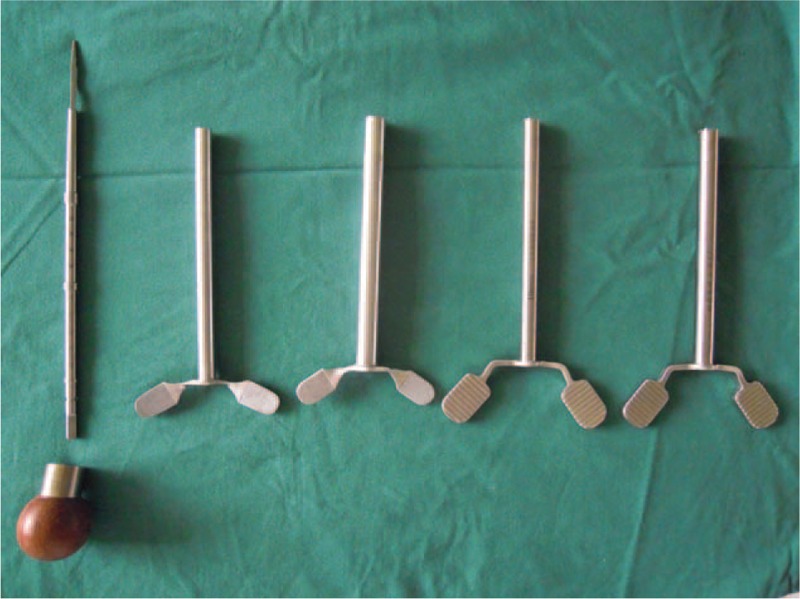
The length of the trephine is about 10 cm, and there are different inner diameters of trephine, ranging from 0.6 cm to 1 cm.

**FIGURE 4:**
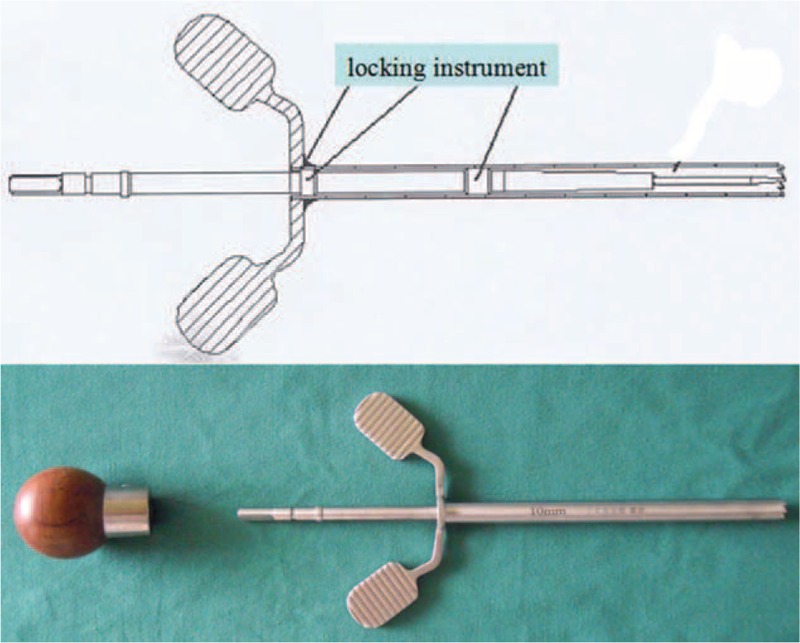
When the serrated top of the modified trephine reaches the tip of the probe, the locking instrument on the probe would restrict the trephine.

Trephine, a smooth cannular instrument with annular wavy serrated top, smooth cylindrical wall, and cross handle, is usually used in anterior cervical corpectomy and fusion (ACCF). The majority of cervical vertebrae can be harvested by trephine at 1 manipulation, displaying high osteotomy efficiency (Fig. 1). However, if the serrated top of the trephine touches the spinal cord, the neurological injury is terrible. When the top reaches the posterior cortical bone, stopping screwing the trephine is proper, but requires experience and skill. For the safety consideration, we added locking instruments on both the trephine and the probe (Fig. 2). The length of the trephine is 10 cm, and there are different inner diameters of trephine, ranging from 0.6 cm to 1 cm (Fig. 3). The modified trephine and probe have been registered in China (the Patent number: ZL 2012 2 0001559.2). When the top of the modified trephine reaches the tip of the probe, the locking instrument on the probe can lock the trephine to avoid the neurovascular injury (Fig. 4).

To the best of our knowledge, no study focuses on the application of trephine in the osteotomy procedure for thoracolumbar kyphotic deformity correction. In this study, we applied the modified trephine in PSO, and aimed to explore the advantage of the modified trephine over high-speed drill as osteotomy instrument.

## MATERIALS AND METHODS

### Patients

The study was approved by Ethics Committee of The Third Hospital of HeBei Medical University from the very beginning. The inclusion criteria were as follows: diagnosis of severe thoracolumbar kyphosis caused by old compressive vertebrae fracture (duration from fracture to surgery for more than 3 years); severe pain at the thoracolumbar region; and conservative treatment for more than 3 months which did not work (bed rest, oral pain medication, physical therapy, etc.). Exclusion criteria were s follows: serious underlying disease (myocardial infarction, cerebral hemorrhage, and cerebral embolism); spinal tumor, tuberculosis, metabolic diseases, and osteoporosis; and multisegmental spinal fracture. All patients were provided written informed consent to participate in this study before the enrollment.

**FIGURE 5:**
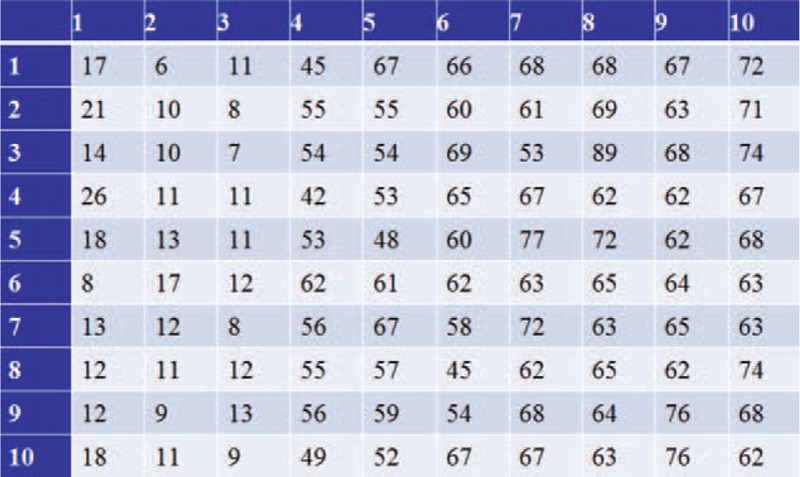
The random number table.

From February 2009 to 2013, 50 patients with severe thoracolumbar kyphotic deformity were prospectively reviewed. All of the patients were randomly assigned to the experimental group (patients received PSO with the modified trephine) and the control group (patients received PSO with high-speed drill), according to random number table method. First, a random number table was created in the computer (Fig. 5). Second, every patient, enrolled in this study, was asked to choose a number within the table, in a way of row (1 to 10) and column (1 to 10); then they were assigned the corresponding numbers. For example, if a patient chose row 6 and column 6, then the corresponding number was 62. Third, if the selected number was odd, the patient was enrolled into the experimental group. If the patient selected an even number, he/she was enrolled into the control group.

**FIGURE 6:**
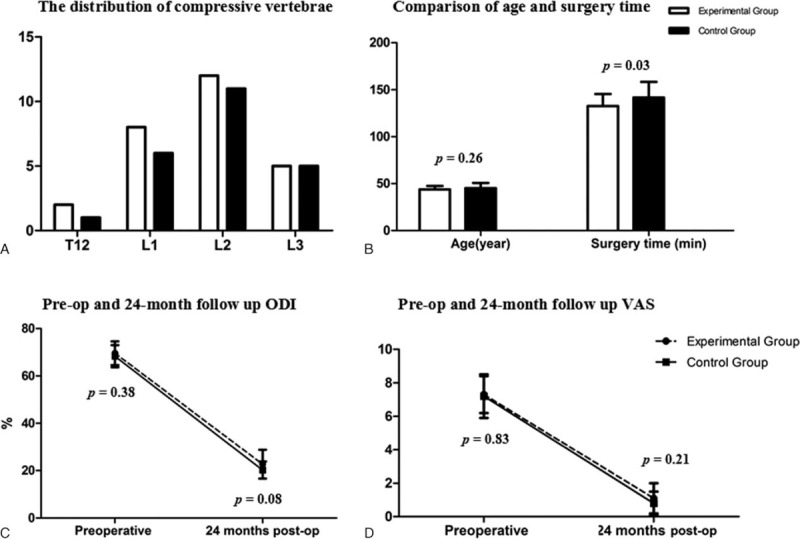
A, The distribution of compressive vertebrae in the experimental group and the control group. B, Comparison of age and surgery time between the experimental group and the control group. C, Preop and 24-month follow-up ODI. D, Preop and 24-month follow-up VAS. ODI = Oswestry Disability Index, VAS = visual analog scale.

**FIGURE 7:**
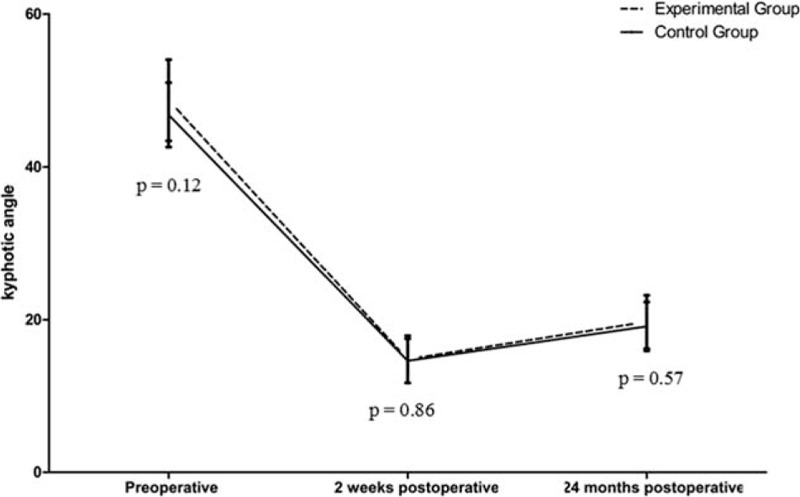
Comparison of preoperative, 2 weeks postoperative, and 24 months postoperative kyphotic angle between the 2 groups.

There were 27 patients who received PSO with modified trephine and were enrolled in the experimental group (17 male and 10 female), and the mean age at the time of surgery was 43.8 ± 3.6 years (range 38–52 y). The distribution of vertebral compression fracture was as follows: 2 patients with T_12_, 8 patients with L_1_, 12 patients with L_2_, and 5 patients with L_3_. There were 23 patients who received PSO with high-speed drill and who were enrolled in the control group (15 male and 8 female), and the mean age of the patients was 45.2 ± 5.4 years (range 36–57 y). The distribution of vertebral compression fracture was as follows: 1 patient with T_12_, 6 patients with L_1_, 11 patients with L_2_, and 5 patients with L_3_ (Fig. 6a). The 2 groups were compatible in age, sex composition, preoperative kyphotic angle, Oswestry Disability Index (ODI), and visual analog scale (VAS) (Table 1, Fig. 6B–D, and Fig. 7).

**TABLE 1:**
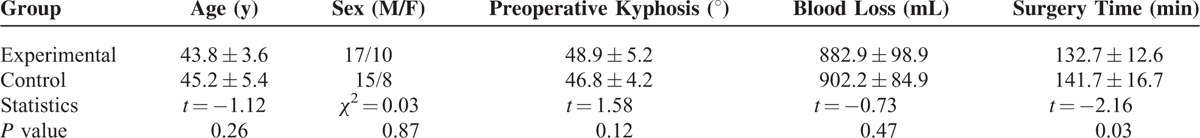
Comparison of General Data and Surgery Data Between Experimental and Control Group

### Surgery Technique

All surgeries were performed by the same combined surgical and anesthesia team. The patient was placed in a prone position; both the motor and somatosensory-evoked potentials (MEPs and SSEPs) were used in all the patients. A standard posterior exposure of the spine was provided and pedicle screws were inserted 2 levels above and below the target vertebrae under C-arm guidance. A laminectomy was performed before the resection of the anterior column. The screws were connected on 1 side with a temporary stabilizing rod, which was contoured to the shape of the deformity. Careful subperiosteal dissection was carried out on the contralateral side (opposite to the stabilizing rod), following the lateral wall of the vertebral body, until the anterior aspect was reached.

### Experimental Group

**FIGURE 8:**
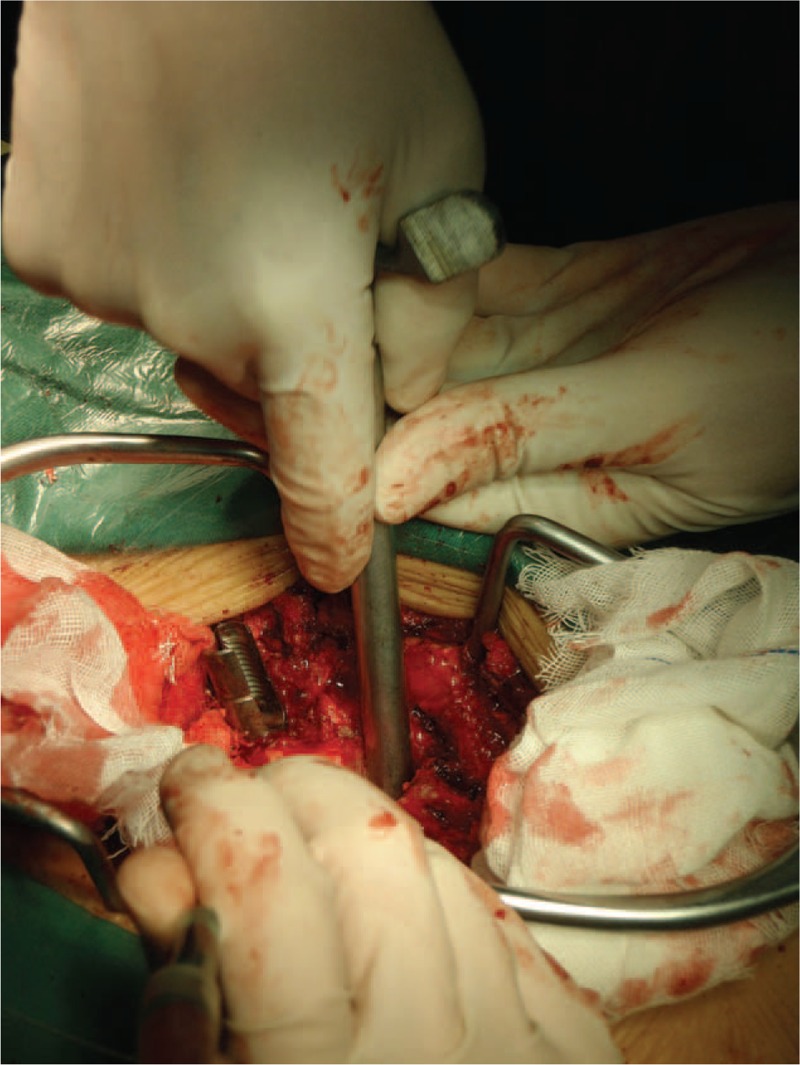
Intraoperative view of the modified trephine being introduced into the vertebrae through the probe.

**FIGURE 9:**
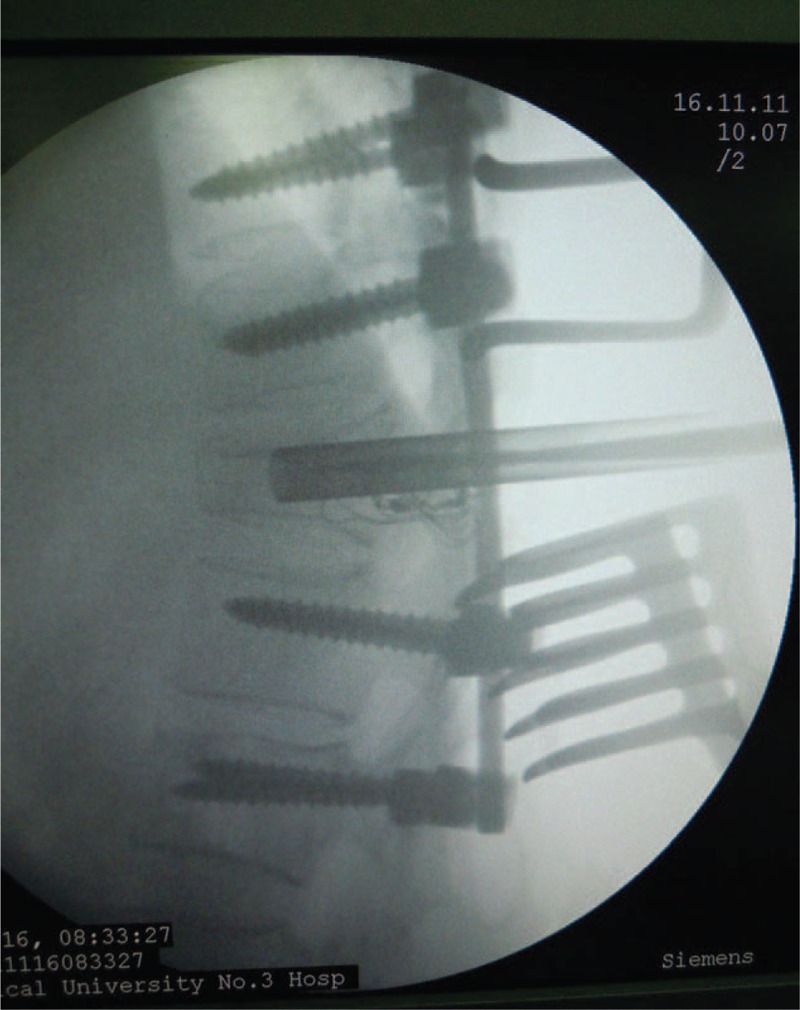
Intraoperative X-ray of the modified trephine being introduced into the vertebrae through the probe.

**FIGURE 10:**
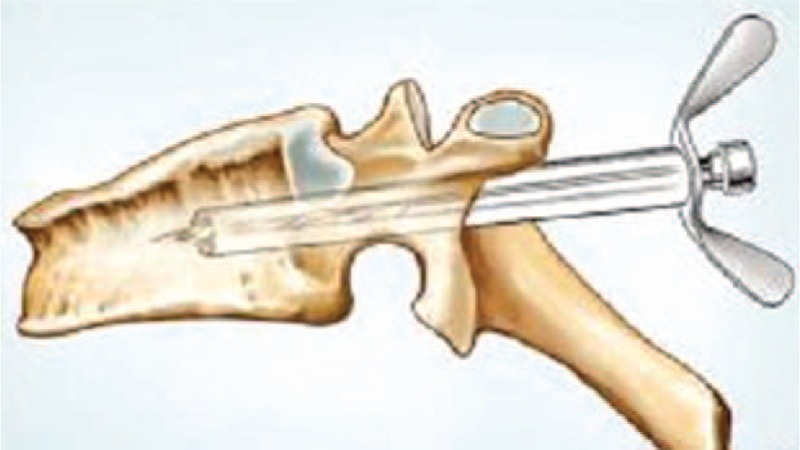
Diagram illustration of the modified hand-held trephine being introduced into the vertebrae.

**FIGURE 11:**
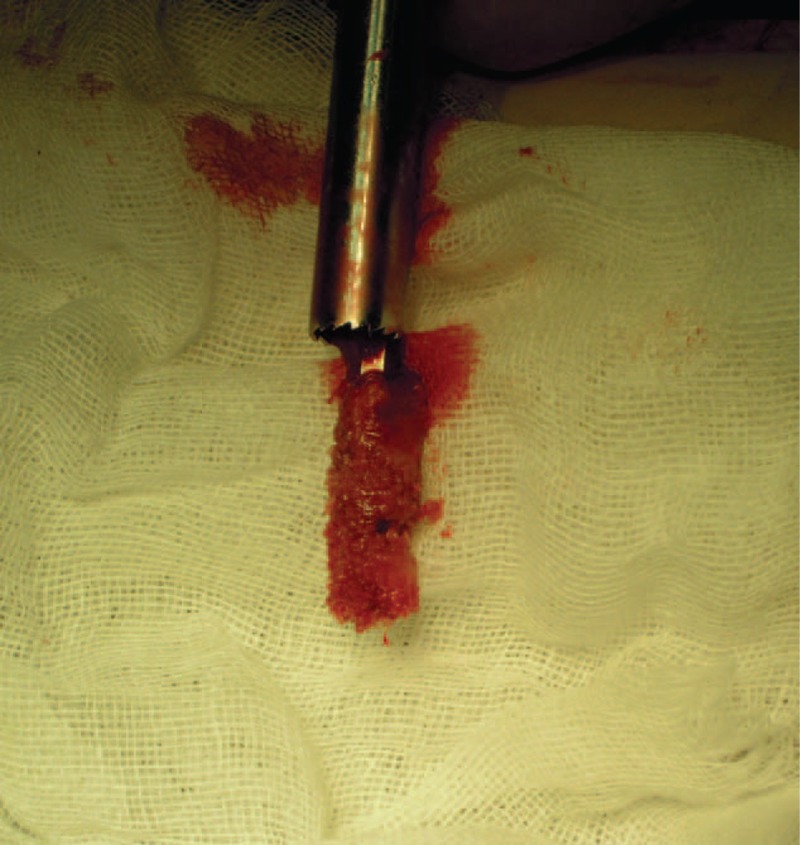
A cylindrical cancellous bone is harvested after the trephine and probe are taken out.

**FIGURE 12:**
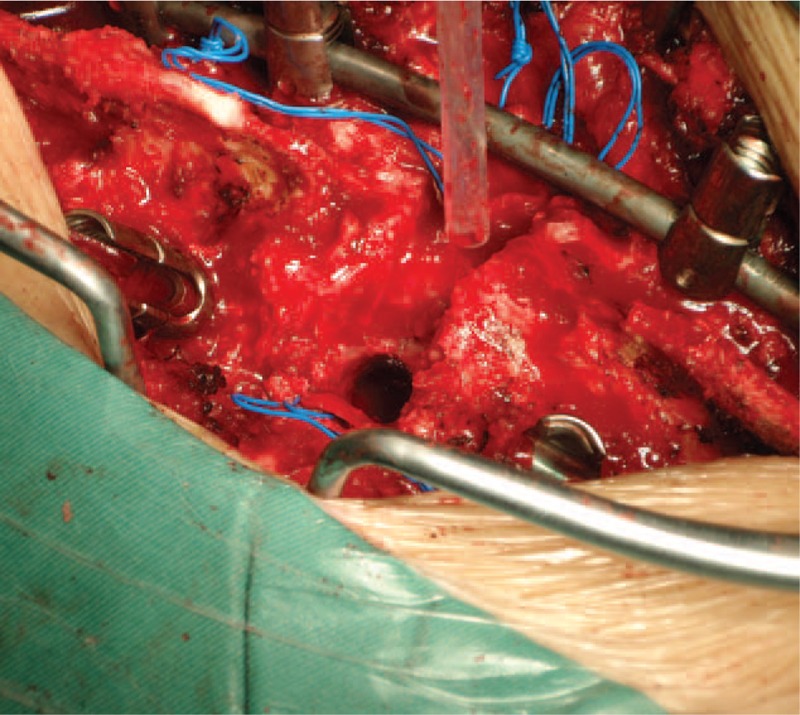
A round osteotomy space is left within the vertebrae after taking out the trephine.

**FIGURE 13:**
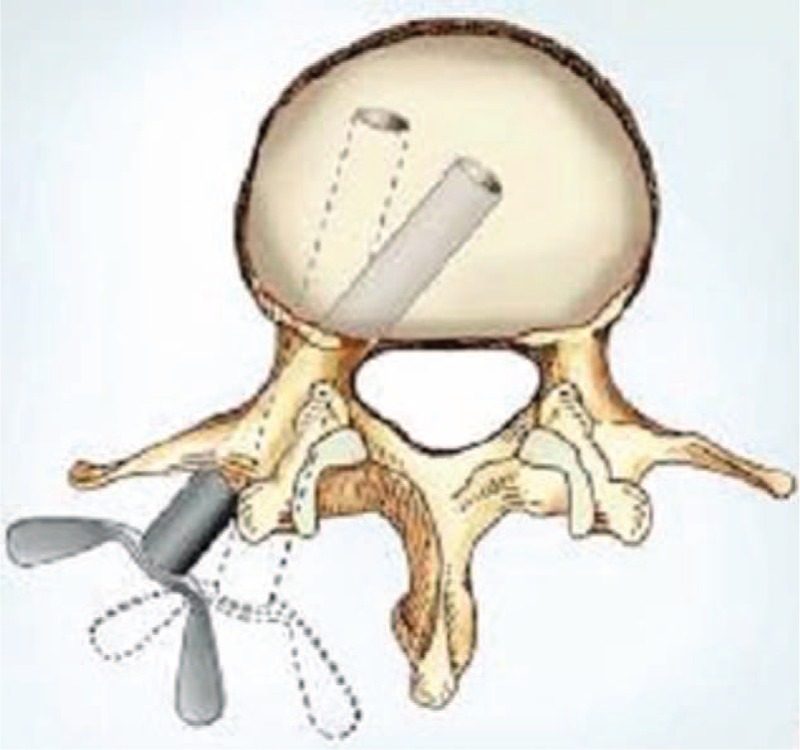
The osteotomy procedure by trephine can be performed from different directions.

**FIGURE 14:**
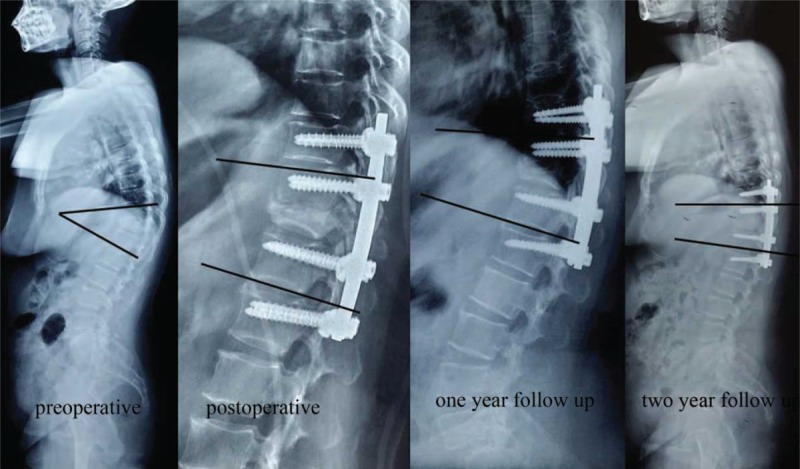
The preoperative kyphotic angle was 39°, the kyphotic angle was 9° at 2 weeks postoperative, 11° at 1-year follow-up, and 12° at 2 years follow-up.

The PSO procedure was performed with the modified trephine. Firstly, the pedicle probe was inserted into the compressed vertebrae under C-arm guidance; then a modified hand-held trephine was introduced into the vertebrae through the probe. Osteotomy could be easily done by twisting the handle of trephine slowly into the vertebrae (Figs. 8–10). When the serrated top of the trephine reached the tip of the probe, the restricted instrument on the probe would block the trephine and prevent the potential injury to the major or radicular vessels ahead. A cylindrical cancellous bone could be harvested after the trephine and probe were taken out together (Fig. 11), with a round space left within the vertebrae (Fig. 12). This procedure could be performed repeatedly, as trephine was inserted into the vertebrae from different directions (Fig. 13). The pedicle of the compressed vertebrae and its lateral wall were removed by curette. An osteotomy procedure on opposite side could be performed in the same manner, to create a wedge osteotomy space. A thin shell of the posterior wall bone anterior to the dural sac was left, and was removed with a rongeur (for a typical case see Fig. 14).

### Control Group

The PSO procedure was performed with high-speed drill. For the compressed vertebrae, a probe was used to determine the entry point and depth. After enlarging the entry with a curette, a 5-mm drill was used to perform regular “egg shell” technique with icy water flush. During this procedure, the inner wall of the pedicle was kept as intact as possible; the cancellous bone from pedicle and vertebral body was cleared thoroughly, till the residual bony cortex and the shell of endplates remain. The lateral wall of the vertebrae and the remaining posterior vertebral wall attached to the posterior longitudinal ligament (PLL) were removed with rongeur on both sides, decompressing the cord circumferentially.

**FIGURE 15:**
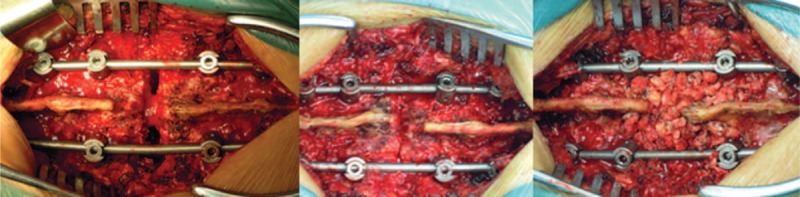
After closure of the osteotomy, bone grafts are placed in the laminae.

Contoured rods were placed bilaterally, sequential compression of the rods was performed to close the osteotomy site posteriorly, till the bone elements touched each other. The closure of the osteotomy should be performed gradually, and under close visualization of the dura, to prevent dural buckling. After closure of the osteotomy space, bone grafts were placed in the laminae (Fig. 15). In the experimental group, the graft was collected from the vertebral bone that harvested by trephine. Whereas in the control group, the high-speed drill ground the cancellous bone to debris, little bone was left for grafting, and autologous bone harvested from ilium was required. Adequate hemostasis was ensured and the wound was thoroughly irrigated with saline. Closed suction drains were inserted at the resection sites and the surgical wound was closed layer-by-layer. Hypotensive anesthesia and perioperative auto transfusion by means of cell-saver were used in all cases to reduce the need for homologous bank blood. Antibiotics were used for all patients on the first postoperative day. Analgesics were used based on patient requirement. The loss through the drains was measured and recorded everyday, the drain was removed when the blood loss through drain was less than 50 mL per 24-hour period. Patients were allowed out of bed at the second postoperative week, with a custom-made plastic thoracolumbosacral orthosis (TLSO), and the TLSO was kept for at least 3 months.

### Effect Evaluation

The clinical records were reviewed for surgery time, blood loss, pain relief, and functional improvement. Preoperative and postoperative pain assessments at the thoracolumbar region were conducted using the (pain scores of 0–10). The ODI was used to make a comprehensive and systematic evaluation of the overall physical condition of the patient at 1 day preoperation and at 24 months postoperation.

### Imaging Evaluation

Standard radiographic measurement was made from standing posterior–anterior and lateral radiographs. The angle of the deformity was measured using lines projected from the upper border of the vertebra above and lower border of the vertebra below the compressed vertebrae. Preoperative and postoperative (2 weeks and 24 months) lateral views were prepared to assess deformity correction (preoperative kyphotic angle-kyphotic angle at 2 wks postoperative) and loss of correction (kyphotic angle at 24 mos postoperative–kyphotic angle at 2 wks postoperative). Radiographic fusion was determined to be solid if there was bone bridging at the osteotomy site in posterior–anterior and in lateral projections.

### Statistical Analysis

Data were analyzed using Statistical Product and Service Solutions software (version 13; SPSS, Chicago, IL). The independent *t* test was used to evaluate numeric variables (age, preoperative kyphotic angle, surgery time, blood loss, correction of kyphotic angle, loss of correction, decrease of ODI, and decrease of VAS), and chi-square test was used to evaluate countable variable (sex composition). Statistical significance was accepted at the 0.05 alpha level.

## RESULTS

**FIGURE 16:**
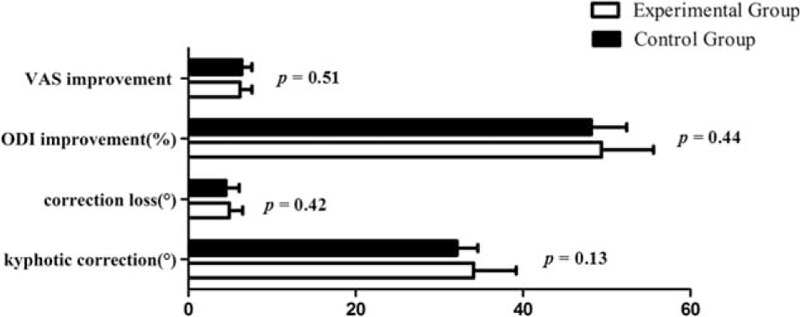
Comparison of VAS improvement, ODI improvement, correction loss, and kyphotic correction between the 2 groups. ODI = Oswestry Disability Index, VAS = visual analog scale.

The surgery time was shorter in the experimental group than that in the control group (132.7 ± 12.6 vs 141.7 ± 16.7 min; *P* = 0.03) (Table 1 and Fig. 6B). No significant difference was found in blood loss (882.9 ± 98.9 mL vs 902.2 ± 84.9 mL; *P* = 0.47) or correction of the kyphotic angle (33.4 ± 3.4° vs 32.1 ± 2.5°; *P* = 0.13) postoperatively between the 2 groups (Tables 1 and 2, Figure 16). At 24-month follow-up, no difference was discovered in loss of the correction (4.9 ± 1.6° vs 4.5 ± 1.6°; *P* = 0.42), change of ODI (49.4 ± 6.2% vs 48.2 ± 4.2%; *P* = 0.44), or in back pain relief (6.2 ± 1.4 vs 6.4 ± 1.2 min; *P* = 0.51) between the 2 groups (Tables 2 and 3, Fig. 16).

**TABLE 2:**
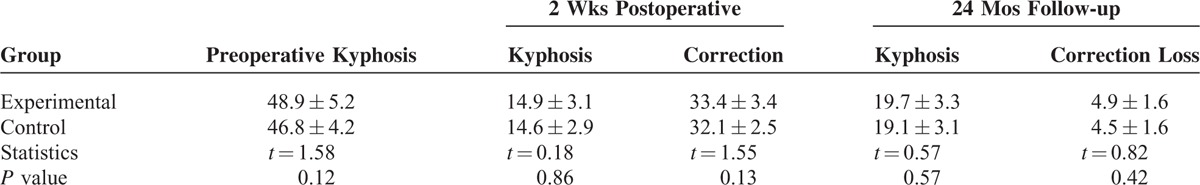
Comparison of Kyphotic Correction and Correction Loss Between the Two Groups (°)

**TABLE 3:**
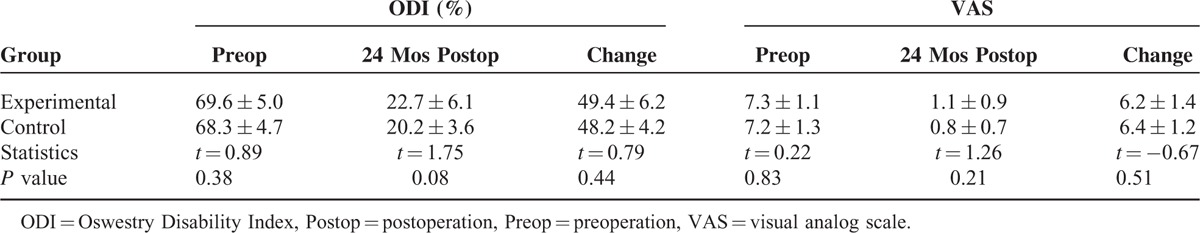
Comparison of ODI and VAS Improvement Between the 2 Groups

There was no implant related complication in this study; bony fusion was detected on lateral X-ray in all patients at 24-month follow-up. In the control group, 2 patients had transient nerve root deficits, but had recovered completely 6 weeks after having undergone the operation, without any intervention. Fourteen patients at 3-month follow-up and 3 patients at 24-month follow-up experienced graft donor site morbidity, and treatment with pain killer medicine was always required (Table 4).

**TABLE 4:**
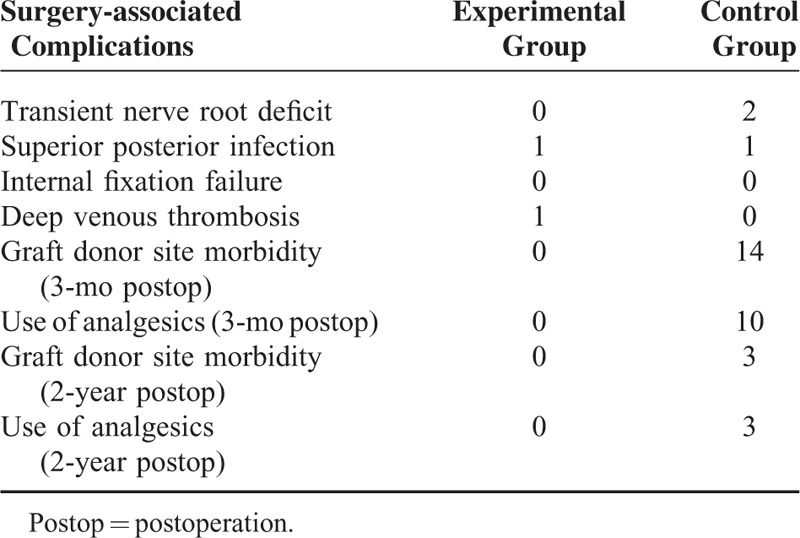
Surgery-associated Complications in the Experimental and Control Group

## DISCUSSION

The key steps of PSO procedure by the modified trephine is summarized as follows:

1. The pedicle probe is inserted into the compressed vertebrae through pedicle, then the modified hand-held trephine is introduced into the vertebrae through the probe without a need for nerve root traction, because the smooth cylindrical wall of the modified trephine presents no threat to the dural sac and the nerve root and the pedicle probe plays the guiding role in this procedure.
2. Osteotomy could be easily performed by twisting the handle of the trephine slowly into the vertebrae. The restricted instrument on the probe can block the trephine when the serrated top of the trephine reaches the tip of the probe, the pedicle probe plays the restrictive/locking role in this procedure.
3. A cylindrical cancellous bone can be harvested after the trephine and the probe are taken out together, and the osteotomy procedure mentioned above can be performed repeatedly as trephine is inserted into vertebrae from different directions.
4. The pedicle of the compressed vertebrae and its lateral wall are removed by curette; a thin shell of the posterior wall bone anterior to the dural sac is removed with a rongeur. After closure of the osteotomy site, bone grafts collected from the harvested vertebral bone by the modified trephine are placed in the laminae.

High-speed drill is commonly used in spinal osteotomy procedure due to its advantages of security and convenience.^22–25^ However, there are two principal defects of using a high-speed drill in PSO. First, after closure of the osteotomy space, bone grafts need to be placed in the laminae to achieve fusion. High-speed drill grinds the cancellous bone within the pedicle and vertebrae to debris, which may lead to heterotopic ossification. Even worse, no harvested bone from the vertebrae is left for grafting. Autologous bone harvested from ilium is required, and the graft donor site morbidity is inevitable. In the control group, 14 patients at 3-month follow-up and 3 patients at 24-month follow-up complained of pain around the graft donor site, and pain killer medicine was always required for these patients. Second, a large amount of heat may be produced when the drill works within the vertebrae, resulting in a thermal burn threat to the spinal cord and nerve root through bone conduction. Icy water flush is required in the procedure to create the wedge osteotomy space with the use of a high-speed drill. The relative fuzzy surgical field, mixed with bone debris, blood, and icy water, may increase the risk of neurovascular injury unintentionally.

One advantage of the modified trephine over high-speed drill is the decrease of surgery time. To complete the wedge shape osteotomy, manipulation of trephine was often needed to be performed 3 or 4 times, with half a minute per time. In other words, less than 5 minutes is enough to finish the osteotomy procedure within the target vertebrae. Whereas in the control group, the relatively complicated manipulation of high-speed drill (icy water flush, fuzzy surgical field) and the extra surgical procedure of autologous bone grafts from ilium increase the overall surgery duration. Another advantage is that the cylindrical vertebral bone harvested by trephine is an ideal graft, and there is no need for autologous bone grafts from ilium, which effectively shortens the duration of surgery, decreases blood loss, and prevents graft-donor site morbidity. The size of harvested bone differs from 0.6 cm × 0.6 cm × 2 cm to 1 cm × 1 cm × 2 cm, depending on the diameter of trephine in use and the depth of trephine being screwed into the vertebral body. Considering the safety of the probe and trephine we modified is solid, risk of nervous impingement and vascular injury in osteotomy procedure is much lower than the use of traditional trephine, and the learning curve for use of the modified trephine is short.

Although it has some advantages, the modified trephine alone is not enough to finish the osteotomy procedure. First, the trephine has the power to harvest vertebral cancellous bone, but is unable to remove cortical bone; this is particularly true regarding the posterior cortical bone anterior to the dural sac. During the osteotomy procedure, the pedicle and lateral wall of the compressed vertebrae are removed by curette, and the posterior wall bone is removed by rongeur. Second, the trephine is a cylindrical instrument, the osteotomy interface created by trephine within the compressed vertebrae is irregular, and further procedure to smoothen its surface before closing the osteotomy space is necessary to achieve bony fusion. In conclusion, the modified trephine and high-speed drill used in the PSO procedure demonstrate comparable radiographic and clinical results. Application of the modified trephine obviously shortens surgery time when compared to the high-speed drill. The cylindrical vertebral bone harvested by trephine is an ideal graft, without the need of autologous bone grafts from ilium, effectively preventing the graft donor site morbidity.
